# How to select and understand guidelines for patient-reported outcomes: a scoping review of existing guidance

**DOI:** 10.1186/s12913-024-10707-8

**Published:** 2024-03-13

**Authors:** Takako Kaneyasu, Eri Hoshino, Mariko Naito, Yoshimi Suzukamo, Kikuko Miyazaki, Satomi Kojima, Takuhiro Yamaguchi, Takashi Kawaguchi, Tempei Miyaji, Takako Eguchi Nakajima, Kojiro Shimozuma

**Affiliations:** 1 College of Life Sciences, Department of Biomedical Sciences, Ritsumeikan University, 1-1-1, Noji-Higashi, Kusatsu, Shiga 525-8577 Japan; 2 Comprehensive Unit for Health Economic Evidence Review and Decision Support, Research Organization of Science and Technology, Ritsumeikan University, Kyoto, Japan; 3 Division of Policy Evaluation, Department of Health Policy, Research Institute, National Center for Child Health and Development, Tokyo, Japan; 4 Department of Oral Epidemiology, Graduate School of Biomedical and Health Sciences, Hiroshima University, Hiroshima, Japan; 5 Tohoku University Graduate School of Medicine, Miyagi, Japan; 6 Department of Health Informatics, School of Public Health, Kyoto University, Kyoto, Japan; 7 Division of Biostatistics, Tohoku University Graduate School of Medicine, Tohoku University, Miyagi, Japan; 8 Department of Practical Pharmacy, Tokyo University of Pharmacy and Life Sciences, Tokyo, Japan; 9 Research Center for Advanced Science and Technology, The University of Tokyo, Tokyo, Japan; 10 Department of Early Clinical Development, Kyoto University Graduate School of Medicine, Kyoto, Japan

**Keywords:** Patient-reported outcomes, Recommendation, Checklist, Handbook, Clinical outcome assessment

## Abstract

**Background:**

Over the past few decades, patient-reported outcomes (PROs) have been used to understand patient health conditions better. Therefore, numerous PRO measures (questionnaires) and guidelines or guidance have been developed. However, it is challenging to select target guidance from among the many available guidance and to understand the chosen guidance. This study comprehensively collected the existing PRO guidance for clinical trials or studies and practices to support novice PRO users in academia, industry, clinical practice, and regulatory and reimbursement decision-making.

**Methods:**

For the scoping review, we searched the MEDLINE, Embase, Google Books, WorldCat, and the National Library of Medicine (NLM) Bookshelf databases from 2009 to 2023. The eligibility criteria were PRO guidance for clinical trials, clinical practice, or application such as health technology assessment. Those guidance cover aspects such as quality of life (QOL), PRO, health-related QOL, health state utilities, psychometric requirements, implementation methods, analysis and interpretation, or clinical practice applications. After the systematic search, three researchers individually reviewed the collected data, and the reviewed articles and books were scrutinized using the same criteria.

**Results:**

We collected the PRO guidance published in articles and books between 2009 and 2023. From the database searches, 1,455 articles and 387 books were identified, of which one book and 33 articles were finally selected. The collected PRO guidance was categorized into the adoption of PRO measures, design and reporting of trials or studies using PROs, implementation of PRO evaluation in clinical trials or studies or clinical practice, analysis and interpretation of PROs, and application of PRO evaluation. Based on this categorization, we suggest the following for novices: When selecting guidance, novices should clarify the “place” and “purpose” where the guidance will be used. Additionally, they should know that the terminology related to PRO and the scope and expectations of PROs vary by “places” and “purposes”.

**Conclusions:**

From this scoping review of existing PRO guidance, we provided summaries and caveats to assist novices in selecting guidance that fits their purpose and understanding it.

**Supplementary Information:**

The online version contains supplementary material available at 10.1186/s12913-024-10707-8.

## Background and introduction

Patient self-assessments have been used in various situations as a tool to understand patients’ health conditions (e.g., pain [1], fatigue [2], anxiety [3]). Numerous measures (questionnaires) [4, 5] and guidelines or guidance [6–8] have been developed and published. The term patient-reported outcome (PRO) was initially defined as the outcome of clinical trials that tested the efficacy and safety of pharmaceuticals [8, 9] but is now widely used in clinical practice [7, 10, 11].

The US Food and Drug Administration (FDA) published guidance for the use of PROs in clinical trials in 2009 [12] and 2014 [13], followed by the Patient-Focused Drug Development Guidance Series [14] around 2020. The European Medicines Agency (EMA) published the PRO guideline for the evaluation of anticancer drugs [15] in 2016 and the International Council for Harmonisation of Technical Requirements for Pharmaceuticals for Human Use (ICH) finalized the Guidance E8 (R1) [16] in 2021.

The US guidances adopted the phrase “clinical outcome assessment (COA)”, which is defined as a superordinate concept of PROs and non-PROs, such as clinician-reported outcomes (ClinRO) [13, 17]. However, the published EMA guideline [15] and the ICH guidance E8 (R1) [16] does not include COA or ClinRO. PROs measured in clinical trials have been consolidated in systematic reviews and clinical practice guidelines to facilitate clinical decision-making. However, in the guideline of systematic review for PRO reports [10, 18], the term clinical outcome set (COS) is used whereas the term COA is not. These differences in the terminology used in the different documents make it difficult for novices to understand their content. (Henceforth, “guideline”, “guidance”, or others regarding PROs were referred to as “guidance” regardless of the original title.)

PROs measured in clinical trials are also applied in health technology assessment (HTA) and reimbursement decisions [7, 10, 11]. However, the difference between preference-based measures (PBM) [19], the source of quality-adjusted life years in HTA, and PRO in a narrow sense is not clearly stated in the guidance [15] or expressed differently (patient preference ratings, utility measures, or PBM) [12, 15, 20], which can lead to confusion.

In clinical practice, PRO assessment has been recognized as a tool for understanding patients health conditions and is expected to promote patient-centered care [21]. The International Society for Quality of Life Research (ISOQOL) has compiled clinical practice reports into best practices for PRO assessment and published them as a guidance. These include PRO assessment in clinical practice, which improves patient-clinician communication and is used for clinical decision-making [20, 22].

Electronic PRO evaluations, collectively called electronic PRO (ePRO), are now widely used in clinical trials [12, 15, 16] and in clinical practice [20], making PRO more accessible.

The expanding use of PROs may cause challenges due to variations in terminology among PRO guidance, differences in PRO scope, and varying expectations (e.g., mere outcomes or more). These discrepancies can pose difficulties for novices seeking PRO guidance in academia, industry, clinical practice, regulatory, and reimbursement decision-making, particularly in selecting appropriate guidance and understanding the content.

This study comprehensively collected and organized the guidance for PRO evaluation from clinical trials to clinical practice to assist PRO novices in selecting and understanding the guidance.

## Method

A scoping literature review was conducted using a search strategy and set of eligibility criteria to examine PRO guidance’s type, target, and purpose. Following the literature search, the experts were directly inquired about the collected guidance information to ensure it was comprehensive. The process followed the Preferred Reporting Items for Systematic reviews and Meta-Analyses extension for Scoping Reviews (PRISMA-ScR) [23].

### Eligibility criteria

First, the documents should be guidance, guidelines, guidebooks, task force reports, recommendations, declarations or etc., related to patient-reported outcomes (PROs); quality of life (QOL), health-related quality of life (HRQL or HRQoL), or health state utilities. Second, the guidance is intended for clinical practice, clinical studies, clinical trials, psychometrics, validation, translation, item response theory, differential item functioning, clinical interpretation, minimum important difference (MID), minimal clinically important difference (MCID), meaningful change, analysis, missing data, ePRO, monitoring, ethics, labeling claims, and health technology assessment (HTA) (For a taxonomy of the above terms, please see Additional file 1). A literature search was anticipated to yield disease-specific, region/country-specific, or race-specific guidance. However, this study did not include these to ensure the generalizability of the search results. As an exception, only oncology- or rheumatology-related PRO guidance with a long history of PRO evaluation and content applicable to other diseases was included in this study.

### Data sources and search strategies

We developed a comprehensive search strategy for academic articles and books in collaboration with an information specialist (KS). Search terms were determined by TK from items addressed in the guidance for clinical trials or studies and clinical practice [12, 14, 20] and books on PRO and QOL [19, 24–26] and were discussed with MN and KS. Given that we anticipated that documents in various formats would be reviewed in electronic or printed form, such as unique monographs or reports, articles in academic journals, and a (series) of chapter(s) in a book, we performed a comprehensive search that included databases that did not focus exclusively on academic publications.

We searched MEDLINE and Embase for academic articles published after 2009 when the FDA PRO guidance was published. We searched Google Books, WorldCat, and the National Library of Medicine (NLM) Bookshelf for books published since the year after the EMA guidance was published in 2016 to reflect updated information in this area. Searches were conducted for MEDLINE and Embase on October 28, 2020, and September 14, 2023;, for WorldCat and the National Library of Medicine Bookshelf on October 22, 2020; and for Google Books on October 25, 2020. WorldCat, the National Library of Medicine Bookshelf, and Google Books were also searched on September 25, 2023 (Additional file 1).

After the systematic search, we emailed members of the ISOQOL Japan Special Interest Group (SY, TY, KT, and MT) to examine the reference lists of the collected studies and determine whether other important PRO guidance was excluded. The resulting candidate guidance were added to the selection process as subsequent documents from other sources.

### Guidance selection

Academic articles were reviewed by three research team members (SK, NM, and KT), and books were reviewed by three (NM, HE, and KT). During the review process, we removed duplicate articles or book information, and the first reviewer screened all citations (title and abstract for articles, and title and table of contents for books) to confirm eligibility for this review. Guidance on technical details (overly narrow in scope) and health system assessment guidance using PRO as one of the datasets (vast in scope) were excluded from this study. A second reviewer screened the citations independently and both reviewers discussed the screening results. If the two reviewers disagreed on the selected article or book, a third reviewer (NM) was involved in the discussion to reach a consensus. All the reviewed articles and books were scrutinized using the same criteria.

### Summary of review results

The collected PRO guidance was categorized by four co-authors (SK, NM, EH, and KT) as follows: adoption of PRO measures, design and reporting of trials or studies using PROs, implementation of PRO evaluation, analysis and interpretation of PROs, and application of PROs. Rather than examining detailed differences in the collected guidance, we focused solely on integrating the information and promoting novices’ understanding.

## Results

### Study selection

**Fig. 1:**
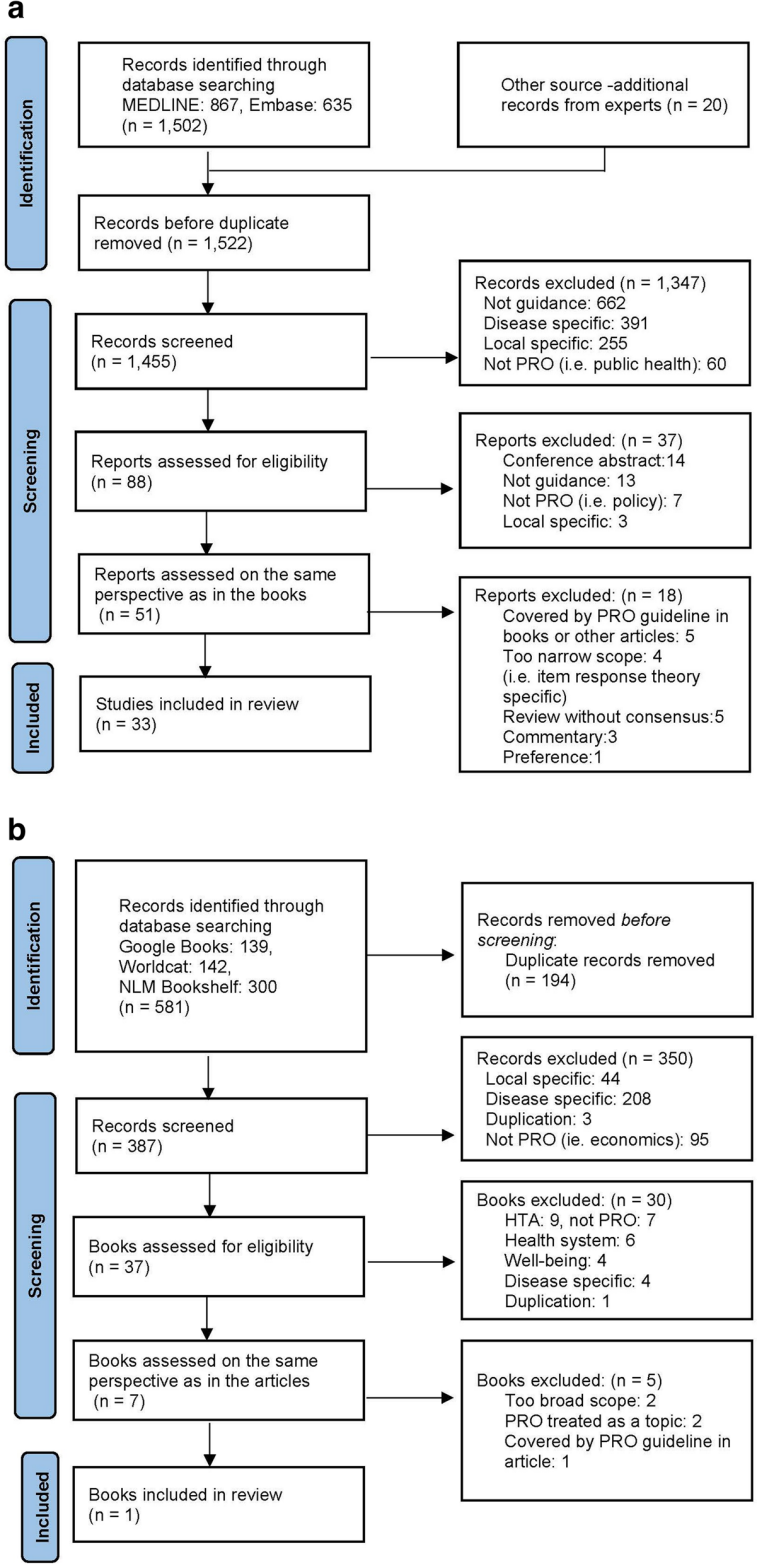
**a** Review of article information, **b** Review of book information

A total of 1,502 articles were identified in the PRO guidance search and 20 additional pieces of information were obtained from experts. After removing the duplicates, 1,522 titles and abstracts were reviewed and refined to 88. After a full-text review, 51 articles met the inclusion criteria. The PRISMA flowchart in Fig. 1a illustrates the process of selecting article information. A total of 581 books were identified and 387 titles and abstracts were selected after duplicates were removed. The full texts of 37 books were reviewed, and six met the inclusion criteria. The PRISMA flowchart in Fig. 1b illustrates the book selection process. They also re-evaluated whether articles and books were selected from the same perspective. Ultimately, information from 33 articles and one book was incorporated into this study.

### Overview of guidance

**Fig. 2:**
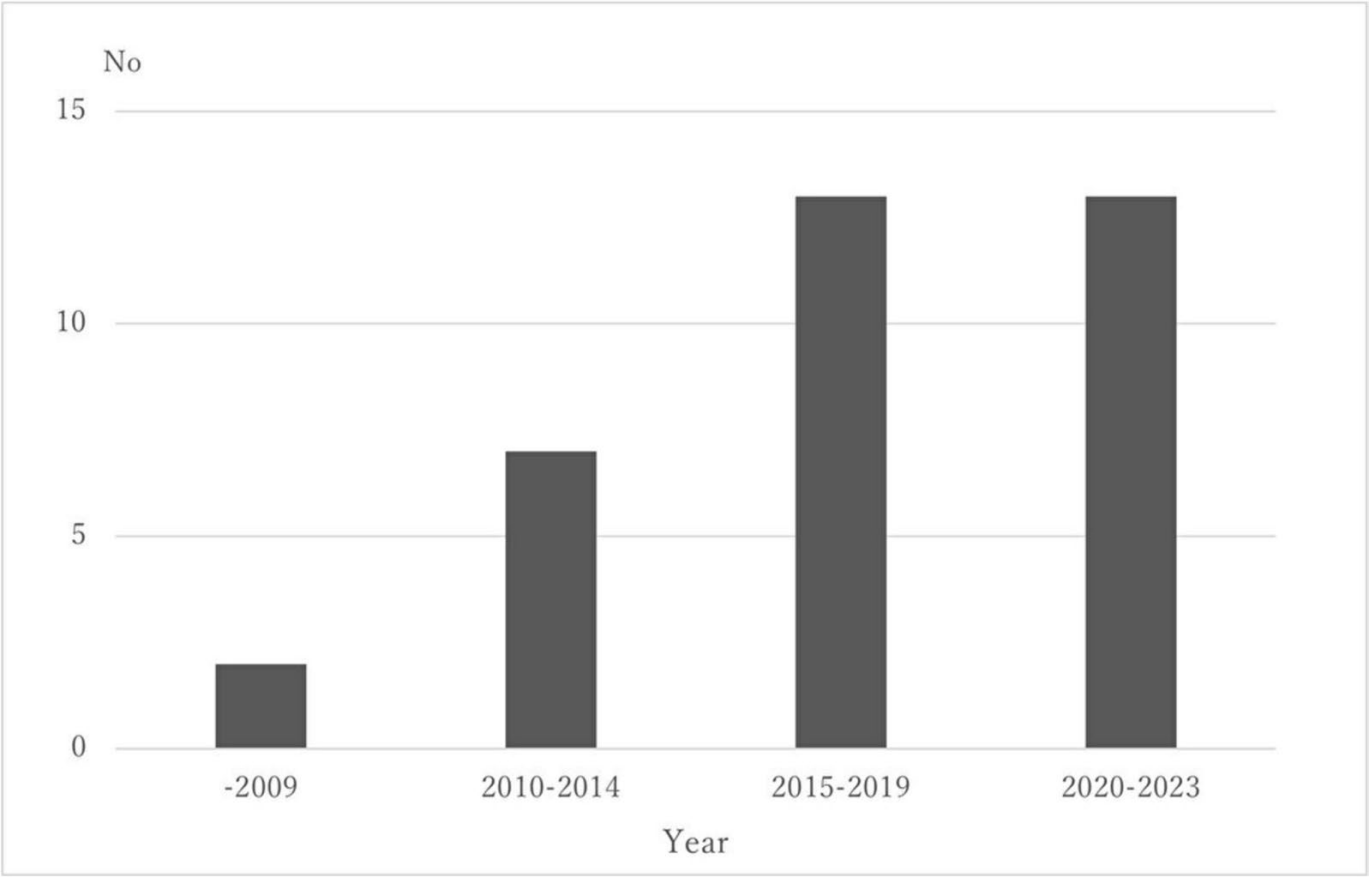
Years of publication

Since the publication of the FDA PRO guidance in 2009 [12], the number of guidance issued has gradually increased (see Fig. 2, Year of Publication). A total of 10 PRO guidance was published from 2009 to 2016, whereas 23 were published in 2017 and beyond, the year after the EMA PRO guidance [15] was issued. Table 1 provides an overview of the articles and books included in this study. The final selected guidance designations were guideline (*n* = 9) [15, 18, 27–33], recommendation (*n* = 8) [34–41], review (*n* = 4) [42–45], guide [46–48], handbook [5, 49, 50], guidance [14, 15, 51] (all *n* = 3), task force report [52, 53], (*n* = 2), checklist [54] and reflection paper [55] (*n* = 1). Regarding guidance specific to PRO evaluation, three were for drug efficacy or safety [14, 15, 51], 11 documents were related to the adoption of PRO measures [5, 14, 15, 30, 32, 34, 35, 38, 45, 49, 55], four were related to the design and reporting of trials/studies [14, 15, 29, 31], seven were related to implementation during PRO evaluation including ePRO and electronic health records [36, 37, 41, 44, 46, 52, 56], and six were related to the analysis and interpretation of PROs [27, 28, 39, 40, 42, 43]. The guidance for the application of PRO was identified as systematic reviews [18, 50], HTA [33, 53], and clinical practice applications [46–48, 51, 54].

**Table 1:** Overview of the articles and books

Title	Year	Author	Purpose	Ref#	Sec #
Articles					13
Patient-reported outcomes: assessment and current perspectives of the guidelines of the Food and Drug Administration and the reflection paper of the European Medicines Agency	2009	Bottomley A, et al.	Differences in the PRO/HRQL Guidance by FDA and EMEA	[42]	NA
Multinational trials - Recommendations on the translations required, approaches to using the same language in different countries, and the approaches to support pooling the data: The ISPOR patient-reported outcomes translation and linguistic validation good research practices task force report	2009	Wild D, et al.	Translation of PRO questionnaire and linguistic validation	[34]	1.2
Evidence-based guidelines for interpreting change scores for the European Organisation for the Research and Treatment of Cancer Quality of Life Questionnaire Core 30	2012	Cocks K, et al.	Interpretation of PRO evaluation results (MCID)	[27]	4.2
Patient-reported outcome measures in safety event reporting: PROSPER consortium guidance	2013	Banerjee Ak, et al.	Use of PRO measures for safety data collection	[51]	5.3.1
Reporting of patient-reported outcomes in randomized trials: The CONSORT-PRO extension	2013	Calvert M, et al.	Description of PRO measurement report	[29]	2
ISOQOL recommends minimum standards for patient-reported outcome measures used in patient-centered outcomes and comparative effectiveness research.	2013	Reeve BB, et al.	Requirements for selecting a PRO measures	[35]	1.3
Methods for interpreting change over time in patient-reported outcome measures	2013	Wyrwich KW, et al.	Interpretation of PRO evaluation results (MCID)	[28]	4.2
Validation of electronic systems to collect patient-reported outcome (PRO) data - recommendations for clinical trial teams: report of the ISPOR ePRO systems validation good research practices task force	2013	Zbrozek A, et al.	Requirements for ePRO systems validation	[52]	3.1
Clinician’s checklist for reading and using an article about patient-reported outcomes.	2014	Wu AW, et al.	Use of PRO assessment articles in clinical practice	[54]	5.3.3
Appendix 2 to the Guideline on the evaluation of anticancer medicinal products in man. The use of patient-reported outcome (PRO) measures in oncology studies.	2016	European Medicines Agency	Use of PRO measures for regulatory submissions in Europe (in cancer clinical trials)	[15]	1, 2, 4.2
How to select outcome measurement instruments for outcomes included in a “Core Outcome Set” - a practical guideline	2016	Prinsen CA, et al.	Core clinical outcome selection	[30]	1.3
Users’ guide to integrating patient-reported outcomes in electronic health records	2017	Snyder C, Wu AW, Ed.	Integrating PRO measures into electronic health records	[46]	3.2
Mapping to estimate health-state utility from non-preference-based outcome measures: An ISPOR good practices for outcomes research task force report	2017	Wailoo AJ, et al.	Mapping from non-preference based measure to utility	[53]	5.2
Articles					13
The COMET Handbook: version 1.0.	2017	Williamson PR, et al.	COS development, implementation, review and feedback	[5]	1.1,
Reflection paper on copyright, patient reported outcome instruments and their translations	2018	Anfray et al.	Copy right for PRO and its interpretation	[55]	1.2
Guidelines for inclusion of patient-reported outcomes in clinical trial protocols: The SPIRIT-PRO extension	2018	Calvert M, et al.	Description of PRO measurement protocol	[31]	2
Standards for instrument migration when implementing paper patient-reported outcome instruments electronically: Recommendations from a qualitative synthesis of cognitive interview and usability studies	2018	Muehlhausen, W, et al.	Requirements for ePRO equivalence assessment	[36]	3.1
COSMIN guideline for systematic reviews of patient-reported outcome measures	2018	Prinsen CAC, et al.	Review methods for PRO evaluation reports	[18]	5.1
Scoping review of response shift methods: current reporting practices and recommendations	2018	Sajobi TT, et al.	Reliability of the PRO questionnaire (response shift)	[43]	4.3
Implementing patient-reported outcome measures in clinical practice: a companion guide to the ISOQOL user’s guide	2019	Chan EKH, et al.	PRO assessment in clinical practice (for diverse uses)	[47]	1.3, 3.2, 5.3
Training on the use of technology to collect patient-reported outcome data electronically in clinical trials: Best practice recommendations from the ePRO Consortium	2019	Ly JJ, et al.	Training ePRO use in clinical trials	[37]	3.1
International standards for the analysis of quality-of-life and patient-reported outcome endpoints in cancer randomized controlled trials: recommendations of the SISAQOL Consortium	2020	Coens C, et al.	Statistical considerations in PRO measurement	[39]	4.1
Translation of patient-reported outcomes in oncology clinical trials to everyday practice	2020	Ivatury SJ, et al.	PRO assessment in clinical practice (from cancer clinical research)	[44]	3.2
Good practices for the translation, cultural adaptation, and linguistic validation of clinician-reported outcome, observer-reported outcome, and performance outcome measures	2020	McKown S, et al.	Considerations for translating the non-PRO questionnaires	[38]	1.2
The OMERACT Handbook. Ver 2.1. 2021.	2021	Beaton D, et al.	Patient engagement in the development of PRO	[49]	1.1
International guidance on the selection of patient-reported outcome measures in clinical trials: a review	2021	Crossnohere NL, et al.	Requirements for selecting a PRO measures	[45]	1.3
COSMIN reporting guideline for studies on measurement properties of patient‑reported outcome measures	2021	Gagnier JJ, et al.	Reporting guideline for the property of PRO measures	[32]	1.3
Articles					7
Using structural equation modeling to investigate change and response shift in patient‑reported outcomes: practical considerations and recommendations	2021	Verdam MGE, et al.	Response shift in PRO evaluation	[40]	4.3
The PROTEUS guide to implementing patient-reported outcomes in clinical practice: A synthesis of resources.	2023	Crossnohere N, et al.	PRO assessment in clinical practice (for systematic use)	[48]	1.3, 3.2, 5.3
EUnetHTA 21 – Individual practical guideline document, D4.4 – OUTCOMES (ENDPOINTS)	2023	EUnetHTA 21	Outcomes in HTA including PRO	[33]	5.2
Best practice recommendations for electronic patient-reported outcome dataset structure and standardization to support drug development	2023	Hudgens S, et al.	Structuring of ePRO datasets incorporating CDISC standards	[41]	3.1
Chapter 18: Patient-reported outcomes, In Higgins J, Thomas J, (Ed.) Cochrane Handbook for Systematic Reviews of Interventions, Version 6.4, 2023	2023	Johnston BC, et al.	PRO in systematic reviews	[50]	5.1
FDA Patient-Focused Drug Development guidance series for enhancing the incorporation of the patient’s voice in medical product development and regulatory decision making	2018–2023	US Food and Drug Administration	Methods for collecting and submitting patient information required for drug approval applications	[14]	1, 2, 4.2
Books					
Electronic patient-reported outcome measures: An implementation handbook for clinical research	2018	Byrom B, Muehlhausen W	Entire landscape of ePROs	[56]	3.1

*CDISC* Clinical Data Interchange Standards Consortium, *COMET* Core Outcome Measures in Effectiveness Trials, *CONSORT* Consolidated Standards of Reporting Trials, *COS* Core outcome set, *COSMIN* The COnsensus-based Standards for the selection of health Measurement Instruments, *EMEA* European Medicines Agency, *ePRO* Electronic PRO, *EUnetHTA* European Network for Health Technology Assessment, *FDA* Food and Drug Administration, *HRQL* Health related quality of life, *HTA* Health technology assessment, *ISPOR* International Society for Pharmacoeconomics and Outcomes Research, *ISOQOL* International Society for Quality of Life Research, *MCID* Minimal clinically important change, *NA* Not applicable, *PRO* Patient-reported outcome, *PROSPER* Patient-Reported Outcomes Safety Event Reporting, *SISAQOL* Setting International Standards in Analyzing Patient-Reported Outcomes and Quality of Life Endpoints Data, *SPIRIT* Standard Protocol Items: Recommendations for Interventional Trials, *Ref#* Reference number, *Sec#* Section number

### Summary of review results

**Fig. 3:**
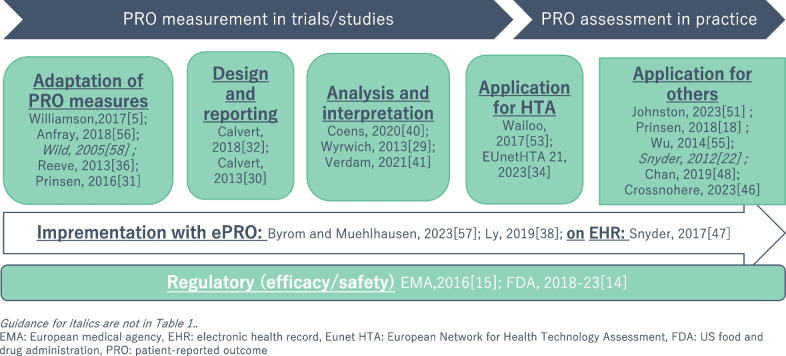
Mapping of guidance for patient-reported outcome from a usage perspective

The collected PRO guidance was categorized into five groups. Figure 3 shows the major categories of guidance. These categories and an outline of guidance are described in detail below.

#### Adoption of patient-reported outcome measures

##### Qualitative research and patient-reported outcome measure development

Identifying outcomes that are important to patients is essential for PRO evaluation [5, 12, 14, 15, 49]. Qualitative research on patient experience has been used for conceptual framework, item development, and content validation in the development of PRO measures [5, 12, 14, 49] (for qualitative research in translation [57], ePRO [36], and MCID [14], see the literature in the respective sections). Interpreting the results of qualitative research requires the support of experts [5], whose cooperation in implementation is essential.

##### Copyright issues and translation

Most PRO questionnaires have been developed and owned by third parties. Therefore, it is essential to ask the questionnaire owner whether translation is possible and obtain licensing and author consent [55]. General guidance for translating PRO questionnaires [57] is also referenced in the guidance of the FDA [14] and EMA [15]. In a multinational clinical trial, there are considerations for its use even when the same questionnaire is used [34]. These translational considerations have also been applied to non-PROs [38].

##### Selection of patient-reported outcome measure

The measurement properties of the PRO measure were established by the COnsensus-based Standards for the selection of health Measurement INstruments (COSMIN) initiative [32]. These are reflected in the following minimum requirements for the selection of PRO measures in clinical trials or studies [35]: 1) conceptual and measurement models, 2) evidence of reliability, 3) content validity, 4) construct validity, 5) responsiveness, 6) score interpretability (see Clinically meaningful differences section), 7) quality of translation, and 8) acceptable burden on patients and investigators. Crossnohere et al. [45] chose these requirements [35] in their review of PRO selection guidance [12, 14, 15, 30].

In clinical practice, the intentions of stakeholders (e.g., clinicians and patients) in identifying outcomes, which are the premise for selecting PRO measures, often diverge [20]. Therefore, the selection of PRO measures necessitates 1) use of existing guidelines and conceptual models, 2) consideration of measurement properties, 3) measurement ease of use, and 4) engagement of clinicians, patients, and other stakeholders to reach a consensus [47, 48, 58].

#### Design and reporting of evaluations using patient-reported outcomes

The endpoints to be assessed by the PROs for clinical trials or studies (e.g., efficacy or safety) should be defined in advance [12, 14, 15], and responder definitions are recommended based on the interpretability of scores (see Clinically meaningful differences section for details) [12, 14, 15]. Reporting [29], and trial protocols [31] standards for clinical trials using PROs (extensions of Consolidated Standards of Reporting Trials (CONSORT) and Standard Protocol Items: Recommendations for Interventional Trials (SPIRIT), see Additional file 2) are also recommended in the regulatory guidance [14, 15].

The purpose of PRO assessment in clinical practice can vary considerably even when this review excludes health system evaluations. Hence, the ISOQOL series of guides [20, 22, 47, 58] emphasizes the need to set goals for PRO assessment, recognize the available resources for conducting the assessment, and strategize how to discuss PRO assessment, specifying when, where, how, and with whom the results will be reported and discussed with patients.

#### Implementation of patient-reported outcomes evaluation

##### ePRO

Byrom and Muehlhausen [56] summarized essential elements of ePRO, including ePRO design, validity considerations in transitioning from paper [36], language processing, ePRO system validation when conducting evaluations [52], user training [37], and “Bring Your Own Device”. The latest ePRO-related information, including the Clinical Data Interchange Standards Consortium (CDISC) standard compliance [41], can be found on the website of the Critical Path Institutes’ PRO Consortium’s Electronic Clinical Outcome Assessment (eCOA) Consortium [59].

##### Patient-reported outcomes assessment in routine clinical practice

The essence of general PRO assessment in clinical practice is summarized in the ISOQOL series of guides [20, 22, 47, 58] and has been adopted in other practice guides [48]. The ISOQOL companion guide [47, 58] addresses issues identified by Ivatury et al. in oncology [44] regarding scale selection, delivery methods, frequency of assessment, and costs and resources in systematic assessment, including ways to address the challenges identified in PRO assessment. In their guidance, Snyder et al. [46] summarizes the strategy, training, evaluation, and administrative, ethical, and legal considerations for integrating PROs into electronic health records.

#### Analysis and interpretation of patient-reported outcome evaluation

##### Statistical methods

The Setting International Standards in Analyzing Patient-Reported Outcomes and Quality of Life Endpoints Data (SISAQOL) Consortium recommendations [39] use cancer clinical trials as examples to categorize the remaining challenges of planning and reporting trials or studies using PROs. These challenges include fit-for-purpose statistical methods, definitions, and management of missing data.

##### Clinically meaningful differences

In regulatory PRO guidance [11, 14, 15], for a reasonable definition of “response” and “worsening” for an individual patient (responder definition in Design and reporting of evaluations using patient-reported outcomes section), a statistical significance test alone is not sufficient. The amount of change or difference obtained must be judged to be MID [28], MCID [28], or a meaningful score difference [14]. The MCID can be used for between-group, within-group, or within-patient changes and requires clarification [28]. Designing clinical trials or studies with a known measure of the MCID facilitates the interpretation of results [15]. Two methods are used to estimate the MCID, one based on anchors and the other based on distributions [11, 14, 28]. Cocks et al. [27] guides sample size calculation and score interpretation in cases where the PRO measure was used for patients with cancer.

##### Response shift

The response shifts are unintended deviations from the PRO measurement results. Sajobi et al. [43] reported that statistical methods for detecting reaction shifts are shifting from then-test methods to structural equation modeling, whereas Verdam et al. [40] conducted modeling to identify response shifts and summarized their interpretation (detection of response shifts and assessment of true changes).

#### Application of patient-reported outcome

##### Systematic review and patient-reported outcomes

The COSMIN initiative promotes high-quality PRO measurement and assessment with guidance for systematic reviews [18] and bias assessment [60]. The Cochrane Handbook for Systematic Reviews of Interventions [50] considers evidence synthesis.

##### PROs in health technology assessment

The EUnetHTA, a network of HTA organizations in Europe, has published guidance for outcomes that include PROs and non-PROs in the context of HTA [33]. However, many clinical trials or studies using PROs do not include PBM to calculate the utility required for HTA and lack relevant preference-based scoring systems. Mapping aims can be used to fill these gaps in evidence. Reporting [61] and methodological [53] guidance is provided for this procedure.

### Patient-reported outcomes in clinical practice

#### Patient-reported outcomes for screening and monitoring

The ISOQOL series of guides [20, 22, 47, 58] lists the best practices that can be used for any purpose, including screening, monitoring, and assessing effectiveness and safety of intervention. This has been incorporated into the Patient-Reported Outcomes Tools, Engaging Users and Stakeholders (PROTEUS) guidance for clinical use [48]. Banerjee et al. [51] proposed a framework for drug safety data collection in pharmaceuticals.

#### Patient-reported outcomes in communication

The significance of PRO assessment (how and why the data are used for treatment) needs to be clearly communicated to improve patient-clinician communication in clinical practice [48]. As described in Patient-reported outcomes for screening and monitoring section, the series of ISOQOL guidance [20, 22, 47, 58] provides best practices for this purpose.

#### Patient-reported outcomes for clinical decision-making

PROs measured in clinical trials can be used for third-party clinical decision making when published as reports. Wu et al. [54] discussed using PRO assessment reports in clinical practice. PRO assessment in clinical practice has created a basis for decision-making by providing patient feedback on the PRO assessment results [20, 22, 47, 58].

## Discussion

Previous exhaustive PRO guidance has been organized regarding PROs for approval, reimbursement, and policy [62]; PROs in clinical trials/studies and clinical practice [48]; and PRO measure utilization [63]. This scoping review collected all guidance except for health system evaluations and organized them into the five sections presented in the results. During this organization, we recognized the need to note the “place” and “purpose” for which guidance is used when choosing and understanding guidance for novice users. The specific sections of this review that should be referred to choose and understand the guidance are identified below.

- Q1: How you can choose among the many types of PRO guidance.
- A1: It is necessary to clarify the “place” and “purpose” where the guidance is used. The guidance that best fits the place and purpose should then be selected. Suppose the purpose is to conduct clinical trials to obtain drug approval in the “drug development” arena. In this case, the guidance listed in Adoption of patient-reported outcome measures, Design and reporting of evaluations using patient-reported outcomes, Implementation of patient-reported outcomes evaluation and Analysis and interpretation of patient-reported outcome evaluation sections, except for Patient-reported outcomes assessment in routine clinical practice section, should be reviewed. If the purpose includes “HTA”, then the guidance in PROs in health technology assessment section should also be reviewed. Furthermore, if the purpose comprises the “development of clinical practice guidelines”, it is advisable to focus on Systematic review and patient-reported outcomes section and review Adoption of patient-reported outcome measures, Design and reporting of evaluations using patient-reported outcomes, Implementation of patient-reported outcomes evaluation and Analysis and interpretation of patient-reported outcome evaluation sections as necessary. If the purpose is to conduct a PRO study in “clinical practice”, the guidance listed in Adoption of patient-reported outcome measures, Design and reporting of evaluations using patient-reported outcomes, Implementation of patient-reported outcomes evaluation and Analysis and interpretation of patient-reported outcome evaluation sections should be consulted first to recognize the differences from routine assessment (Patient-reported outcomes in clinical practice section). For a better understanding of PRO evaluation in routine clinical practice, Patient-reported outcomes in clinical practice section should be consulted first. To obtain an overall picture of PRO evaluation, read the Core Outcome Measures in Effectiveness Trials (COMET) Initiative handbook [5].
- Q2: How you can understand the selected PRO guidance.
- A2: The terminology related to PROs and their scope and expectations vary by “place” and “purpose”. It is advisable to be cognizant of the following differences to understand the guidance better (see Additional file 3 for more detailed definitions).

In clinical trials or studies, what is expected for PROs is the outcome of the trial or study. However, PROs in clinical practice may be expected to serve as communication tools, as indicated in Patient-reported outcomes in communication section, rather than simply outcome.

PROs are used as a measure of health in drug approval and PBM, an indicator of health value, is used in HTA, as described in PROs in health technology assessment section. However, it should be noted that in some countries (e.g., the United Kingdom), PBM may also be referred to as PROs (i.e., the scope of PROs varies).

The terminology associated with PROs varies according to regional and national clinical trial guidance, as noted in the background, and by disease area and application (e.g., systematic reviews). Therefore, when reading the selected guidance, it is advisable first to review the definitions of PROs and their related terms. (Additional file 3 provides examples of synonyms that may be difficult to understand using only a single guidance).

This study has some limitations. First limitation was the keywords setting for the titles of the guidance, which were based on existing guidance and books. However, the titles of the collected guidance were sometimes described as checklists or handbooks. It is possible that adding these terms to the keywords made it more efficient to obtain the desired guidance. Second limitation is that the database used to retrieve article information specializing in the medical sciences did not use PsycINFO in psychology. Therefore, guidance for qualitative research (e.g., COREQ: Consolidated criteria for reporting qualitative research [64] and CIRF: Cognitive Interviewing Reporting Framework [65]) were not included in this review. Although a previous study [35] used psychological databases, consultations with experts yielded more relevant information than database searches. We believe that the comprehensiveness of the present review was ensured by consulting ISOQOL Japan Special Interest Group members. Third limitation is that disease-specific guidance was excluded from the collection. However, a 2013 review by the SPIRIT-PRO group of guidance documents from 1989 to 2013 focused chiefly on HRQL or PRO assessments in cancer clinical trials, and 21,175 reports were screened after removing deduplicates [6]. The inclusion of disease-specific guidance may unnecessarily expand the scope of this review. This study prioritized the feasibility of a comprehensive strategy spanning both scholarly articles and book information. Fourth limitation was the lack of comparison between the series of FDA guidance and other guidance regarding the definition of COA. For example, the FDA’s COA includes patient preference information for medical devices [66]. Although Hollin et al. [67] cited PRO guidance and recommended the validity of preference evidence from qualitative studies, PROs differ from patient preferences, which may confuse novices. Patient preference information was outside the scope of this study, and that article [67] was ultimately excluded. In the future, collecting and organizing guidance for patient preference information may be necessary.

## Conclusions and implications

In this scoping review, existing PRO guidance was categorized into adopting PRO measures, designing and reporting of trials or studies using PROs, implementing PRO evaluation, analyzing and interpreting PROs, and applying PRO evaluation. Based on this categorization, we suggest the following for novices: When selecting guidance, novices should clarify the “place” and “purpose” where the guidance will be used. Additionally, they should know that the terminology related to PRO and the scope and expectations of PROs vary by “places” and “purposes”.

### Supplementary Information

**Supplementary Material 1.**

**Supplementary Material 2.**

**Supplementary Material 3.**

## Data Availability

The literature review data generated in this study are available upon request from the corresponding author.

## Abbreviations

ClinRO: Clinician-reported outcomes
COA: Clinical outcomes assessment
EMA: European Medicines Agency
ePRO: Electronic patient-reported outcome
FDA: US Food and Drug Administration
HTA: Health technology assessment
HRQL: Health related quality of life
ISOQOL: International Society for Quality of Life Research
MID: Minimum important difference
MCID: Minimal clinically important change
PBM: Preference- based measure
PRO: Patient-reported outcome
QOL: Quality of life
