# Supplementary material for: How to select and understand guidelines for patient-reported outcomes: a scoping review of existing guidance
Source: BMC Health Serv Res. 2024 Mar 13;24:334. doi: 10.1186/s12913-024-10707-8 (PMC10938752; doi:10.1186/s12913-024-10707-8)
Supplement: Supplementary file 1 — Supplementary Material 1. [file 12913_2024_10707_MOESM1_ESM.docx]

| **Additional file 1 - Search Strategy**  *Eligibility criteria*  Articles or books must provide guidance that defined as below.  (1) Type of document:  guideline, guidance, guidebook, taskforce report, recommendation, declaration  (2) Target of documents  quality of life (QOL), patient-reported outcome (PRO), health-related quality of life (HRQOL), health-state utilities (3) Purpose of document:  Setting: clinical practice, clinical study, clinical trial  Psychometric requirement: validation, translation, psychometrics, item response theory, differential item functioning,  Clinical interpretation: minimum important difference, meaningful change analysis  Collecting method and analysis: missing data, ePRO, monitoring, ethics  Goal: labelling claims, health technology assessment (HTA)  *Databases:*  For papers: MEDLINE and Embase  For books: Google Books, Worldcat, and National Library of Medicine(NLM) Bookshelf  *Details of strategies:*  Dated on 2020/10/28 and 2023/09/14 |
| --- |
| Search Strategy (MEDLINE / Embase) |
| (MJMESH.EXACT.EXPLODE("quality of life") OR MJEMB.EXACT.EXPLODE("quality of life") OR TI(quality of p/1 life) OR MJMESH.EXACT.EXPLODE("Patient Reported Outcome Measures") OR EMB.EXACT.EXPLODE("patient-reported outcome") OR TI(patient reported p/1 outcome*) OR TI("health-related quality of life") OR TI(hrqol) OR MJEMB.EXACT.EXPLODE("health status") OR MJEMB.EXACT.EXPLODE("health status indicator") OR MJMESH.EXACT.EXPLODE("Health Status") OR MJMESH.EXACT.EXPLODE("Health Status Indicators") OR TI("health state utility") OR TI("health state utilities") OR TI("health utility") OR TI("health utilities")) AND (MJEMB.EXACT.EXPLODE("guideline") OR MJMESH.EXACT.EXPLODE("Guideline") OR MJMESH.EXACT.EXPLODE("Guideline") OR MJMESH.EXACT.EXPLODE("Guidelines as Topic") OR TI(guidance) OR TI(guidebook*) OR TI(taskforce*) OR TI(recommendation*) OR TI(declaration*)) |

*Limits applied_Narrowed by: Entered date: 2009 – 2023

Dated on 2020/10/22 and 2023/09/25

| Search Strategy (Worldcat) |
| --- |
| TI:"health related quality of life" OR KW:"health related quality of life" OR TI:"patient reported outcome" OR KW:"patient reported outcome" OR SU:"patient reported outcome" OR TI:"health state utility"  OR TI:"health state utilities" OR KW:"health state utility" |
| Dated on 2020/10/25 and 2023/09/25 |
| Search Strategy (Google books) |
| "health utility" OR "health utilities" OR "health status indicator"  "hrqol" OR "health related quality of life"  "quality-of-life" OR "qol"  “patient reported outcome“ OR “patients reported outcome“  *Target all test information. |
| Dated on 2020/10/22 and 2023/09/25 |
| Search Strategy (NLM Bookshelf) |
| ("patient reported outcome"[ALL] OR "health utilities"[ALL] OR "quality of life"[ALL]) AND "Health Care Quality, Access, and Evaluation"[MAJR] |
