# Supplementary material for: How to select and understand guidelines for patient-reported outcomes: a scoping review of existing guidance
Source: BMC Health Serv Res. 2024 Mar 13;24:334. doi: 10.1186/s12913-024-10707-8 (PMC10938752; doi:10.1186/s12913-024-10707-8)
Supplement: Supplementary file 2 — Supplementary Material 2. [file 12913_2024_10707_MOESM2_ESM.docx]

| Table 1. CONSORT-PRO: Information for Reporting Randomized Controlled Trials with Patient reported Outcomes | | | |
| --- | --- | --- | --- |
| Section/Topic | Item | CONSORT 2010 Statement Checklist Item | PRO-Specific Extensions Are Prefaced by the letter *P* |
| Title and Abstract | 1b | Structured summary of trial design, methods, results, and conclusions (for specific guidance see CONSORT for abstracts) | P1b: The PRO should be identified in the abstract as a primary or secondary outcome |
| Introduction/Background and objectives | 2a | Scientific background and explanation of rationale | Including background and rationale for PRO assessment |
|  | 2b | Specific objectives or hypotheses | P2b: The PRO hypothesis should be stated and relevant domains identified, if applicable |
| Methods /Participants | 4a | Eligibility criteria for participants | Not PRO-specific, unless the PROs were used in eligibility or stratification criteria |
| Methods/Outcomes | 6a | Completely defined prespecified primary and secondary outcome measures, including how and when they were assessed | P6a: Evidence of PRO instrument validity and reliability should be provided or cited if available including the person completing the PRO and methods of data collection (paper, telephone, electronic, other) |
| Methods/Sample size | 7a | How sample size was determined | Not required for PRO unless it is a primary study outcome |
| Methods/Statistical methods | 12a | Statistical methods used to compare groups for primary and secondary outcomes | P12a: Statistical approaches for dealing with missing data are explicitly stated |
| Results/Participant flow | 13a | For each group, the numbers of participants who were randomly assigned, received intended treatment, and were analyzed for the primary outcome | The number of PRO outcome data at baseline and at subsequent time points should be made transparent |
| Results/Baseline data | 15 | A table showing baseline demographic and clinical characteristics for each group | Including baseline PRO data when collected |
| Results/Numbers analyzed | 16 | For each group, number of participants (denominator)included in each analysis and whether the analysis was by original assigned groups | Required for PRO results |
| Results/Outcomes and estimation | 17a | For each primary and secondary outcome, results for each group, the estimated effect size, and its precision (such as 95% confidence interval) | For multidimensional PRO results from each domain and time point |
| Results/Ancillary analyses | 18 | Results of any other analyses performed, including subgroup analyses and adjusted analyses, distinguishing prespecified from exploratory | Including PRO analyses, where relevant |
| Discussion /Limitations | 20 | Trial limitations, addressing sources of potential bias, imprecision, and, if relevant, multiplicity of analyses | P20/21: PRO–specific limitations and implications for generalizability and clinical practice |
| Discussion /Generalizability | 21 | Generalizability (external validity, applicability) of the trial findings | P20/21: PRO–specific limitations and implications for generalizability and clinical practice |
| Discussion /Interpretation | 22 | Interpretation consistent with results, balancing benefits and harms, and considering other relevant evidence | PRO data should be interpreted in relation to clinical outcomes including survival data, where relevant |

PRO: Patient reported outcome, CONSORT: Consolidated Standards of Reporting Trials

| Table 2. SPIRIT-PRO Extension Checklist: Recommended Items to Address in a Clinical Trial Protocol | | | | |
| --- | --- | --- | --- | --- |
| SPIRIT Section | SPIRIT Item No. | SPIRIT Item Description | SPIRIT-PRO Item No. | SPIRIT-PRO Extension or Elaboration Item Description |
| Administrative Information | | | | |
| Roles and responsibilities | 5a | Names, affiliations, and roles of protocol contributors | SPIRIT-5a-PRO Elaboration | Specify the individual(s) responsible for the PRO content of the trial protocol. |
| Introduction | | | | |
| Background and rationale | 6a | Description of research question and justification for undertaking the trial, including summary of relevant studies (published and unpublished) examining benefits and harms for each intervention | SPIRIT-6a-PRO Extension | Describe the PRO-specific research question and rationale for PRO assessment and summarize PRO findings in relevant studies. |
| Objectives | 7 | Specific objectives or hypotheses | SPIRIT- 7-PRO Extension | State specific PRO objectives or hypotheses (including relevant PRO concepts/domains). |
| Methods: Participants, Interventions, and Outcomes | | | | |
| Eligibility criteria | 10 | Inclusion and exclusion criteria for participants; if applicable, eligibility criteria for study centers and individuals who will perform the interventions (eg, surgeons, psychotherapists) | SPIRIT-10-PRO Extension | Specify any PRO-specific eligibility criteria (eg, language/reading requirements or prerandomization completion of PRO). If PROs will not be collected from the entire study sample, provide a rationale and describe the method for obtaining the PRO subsample. |
| Outcomes | 12 | Primary, secondary, and other outcomes, including the specific measurement variable (eg, systolic blood pressure), analysis metric (eg, change from baseline, final value, time to event), method of aggregation (eg, median, proportion), and time point for each outcome; explanation of the clinical relevance of chosen efficacy and harm outcomes is strongly recommended | SPIRIT- 12-PRO Extension | Specify the PRO concepts/domains used to evaluate the intervention (eg, overall health-related quality of life, specific domain, specific symptom) and, for each one, the analysis metric (eg, change from baseline, final value, time to event) and the principal time point or period of interest. |
| Participant timeline | 13 | Time schedule of enrollment, interventions (including any run-ins and washouts), assessments, and visits for participants; a schematic diagram is highly recommended | SPIRIT- 13-PRO Extension | Include a schedule of PRO assessments, providing a rationale for the time points, and justifying if the initial assessment is not prerandomization. Specify time windows, whether PRO collection is prior to clinical assessments, and, if using multiple questionnaires, whether order of administration will be standardized. |
| Sample size | 14 | Estimated number of participants needed to achieve study objectives and how it was determined, including clinical and statistical assumptions supporting any sample size calculations | SPIRIT- 14-PRO Elaboration | When a PRO is the primary end point, state the required sample size (and how it was determined) and recruitment target (accounting for expected loss to follow-up). If sample size is not established based on the PRO end point, then discuss the power of the principal PRO analyses. |
| Methods: Data Collection, Management, and Analysis | | | | |
| Data collection methods | 18a | Plans for assessment and collection of outcome, baseline, and other trial data, including any related processes to promote data quality (eg, duplicate measurements, training of assessors) and description of study instruments (eg, questionnaires, laboratory tests) along with their reliability and validity, if known; reference to where data collection forms can be found, if not in the protocol | SPIRIT-18a (i)-PRO Extension | Justify the PRO instrument to be used and describe domains, number of items, recall period, and instrument scaling and scoring (eg, range and direction of scores indicating a good or poor outcome). Evidence of PRO instrument measurement properties, interpretation guidelines, and patient acceptability and burden should be provided or cited if available, ideally in the population of interest. State whether the measure will be used in accordance with any user manual and specify and justify deviations if planned. |
|  |  |  | SPIRIT-18a (ii)-PRO Extension | Include a data collection plan outlining the permitted mode(s) of administration (eg, paper, telephone, electronic, other) and setting (eg, clinic, home, other). |
|  |  |  | SPIRIT-18a (iii)-PRO Extension | Specify whether more than 1 language version will be used and state whether translated versions have been developed using currently recommended methods. |
|  |  |  | SPIRIT-18a (iv)-PRO Extension | When the trial context requires someone other than a trial participant to answer on his or her behalf (a proxy-reported outcome), state and justify the use of a proxy respondent. Provide or cite evidence of the validity of proxy assessment if available. |
|  | 18b | Plans to promote participant retention and complete follow-up, including list of any outcome data to be collected for participants who discontinue or deviate from intervention protocols | SPIRIT-18b (i)-PRO Extension | Specify PRO data collection and management strategies for minimizing avoidable missing data. |
|  |  |  | SPIRIT-18b (ii)-PRO Elaboration | Describe the process of PRO assessment for participants who discontinue or deviate from the assigned intervention protocol. |
| Statistical methods | 20a | Statistical methods for analyzing primary and secondary outcomes. Reference to where other details of the statistical analysis plan can be found, if not in the protocol | SPIRIT- 20a-PRO Elaboration | State PRO analysis methods, including any plans for addressing multiplicity/type I (α) error. |
|  | 20c | Definition of analysis population relating to protocol nonadherence (eg, as randomized analysis) and any statistical methods to handle missing data (eg, multiple imputation) | SPIRIT- 20c-PRO Elaboration | State how missing data will be described and outline the methods for handling missing items or entire assessments (eg, approach to imputation and sensitivity analyses). |
| Methods: Monitoring | | | | |
| Harms | 22 | Plans for collecting, assessing, reporting, and managing solicited and spontaneously reported adverse events and other unintended effects of trial interventions or trial conduct | SPIRIT- 22-PRO Extension | State whether or not PRO data will be monitored during the study to inform the clinical care of individual trial participants and, if so, how this will be managed in a standardized way. Describe how this process will be explained to participants; eg, in the participant information sheet and consent form. |

PRO: Patient reported outcome, SPIRIT: Standard Protocol Items: Recommendations for Interventional Trials
