# Supplementary material for: How to select and understand guidelines for patient-reported outcomes: a scoping review of existing guidance
Source: BMC Health Serv Res. 2024 Mar 13;24:334. doi: 10.1186/s12913-024-10707-8 (PMC10938752; doi:10.1186/s12913-024-10707-8)
Supplement: Supplementary file 3 — Supplementary Material 3. [file 12913_2024_10707_MOESM3_ESM.docx]

**Additional file 3 -Glossary**

Measuring health (PRO) or valuing health (PBM)

Patient-related outcomes (PROs) are used for screening, disease management, and clinical trials/studies to evaluate intervention effectiveness and safety. PRO (and health-related quality of life [HRQL], discussed below) is a method that records the health condition of patients (profiles) [1]. Valuations used for health technology are called preference-based measures (PBM), which are a method of weighing (based on preferences) the health condition of patients based on the values of the general population’s health and are different from PRO [2]. However, the implementation of PBM assessment in clinical trials/clinical research is limited, and attempts are being made to bridge (map) the two [3,4].

Definition of PROs and HRQL

In clinical trials/studies, PROs are defined as including health-related quality of life (HRQL) for convenience [5,6]. However, the original definition states that "PROs have aspects that do not overlap with HRQL" [7], and the definition of PROs in clinical practice (e.g., PRO assessment may contribute to improved patient quality of life [8]) is consistent with this original definition. When describing PROs in clinical studies, we need to be careful that the difference between the original definition and the definition for trials does not cause confusion.

Differences between COU, COA and COS

The term concept of use (COU) emerged from discussions within ISPOR (International Society for Pharmacoeconomics and Outcomes Research) and the US Food and Drug Administration (FDA). COU describes the outcome assessment setting and manner of use and is a prerequisite for selecting a clinical outcome assessment (COA) that evaluates clinical benefit [9,10]. Core outcome set (COS) is a set of standard outcomes proposed by COMET (Core Outcome Measures in Effectiveness Trials)/COSMIN (Core Outcome Measures in Effectiveness Trials) and OMERACT (Outcome measures in rheumatology), which provides information for the outcome setting and how to use COS, especially in clinical areas such as rheumatology and pain [7,11]. Currently, qualified COA [12] and COS [13] are shown on their websites. For clinical trials/studies, it is important to select a scale that is consistent with the purpose of the PRO evaluation.

Definition of PROs and non-PROs

In drug development, ISPOR/FDA defines PROs as an element of COA by listing them together with non-PROs, such as clinician-reported outcomes (ClinROs), observer-reported outcomes (ObsROs) and performance outcomes (PerfOs) [9,10,14]. However, the other guidelines, such as those from the European Medical Agency and the ICH (International Council for Harmonization of Technical Requirements for Pharmaceuticals for Human Use), do not mention non-PROs. Canadian discussions [15] have noted that the distinction between PROs and non-PROs is often not straightforward because some constructs can be accurately reported by patients, bypassing the need for clinical assessment. PRO novices should confirm how non-PROs are handled in their respective clinical areas.

Proxy-reported outcome and ObsRO

PRO evaluation in clinical practice as well as in clinical trials/studies can be difficult because patients are unable to express what they feel (e.g., children or patients with dementia). In such cases, the evaluation method may involve a proxy or observer for the patient to compensate for this difficulty. Because ObsRO is more conservative than proxy reporting, the FDA [14] and EMA [6] recommend the ObsRO specification in such cases.

References

1. Fayers, P, & Machin, D. Quality of Life: The Assessment, Analysis and Interpretation of Patient-reported Outcomes, Third edition. (3rd ed., pp. 447-460).
2. Fayers, P, & Machin, D. Quality of Life: The Assessment, Analysis and Interpretation of Patient-reported Outcomes, Third edition. (3rd ed., pp. 3-9).
3. Wailoo AJ, Hernandez-Alava M, Manca A, et al. Mapping to Estimate Health-State Utility from Non-Preference-Based Outcome Measures: An ISPOR Good Practices for Outcomes Research Task Force Report. Value Health.2017;20(1):18-27. doi: 10.1016/j.jval.2016.11.006.
4. Petrou S, Rivero-Arias O, Dakin H, et al. The MAPS Reporting Statement for Studies Mapping onto Generic Preference-Based Outcome Measures: Explanation and Elaboration. Pharmacoeconomics. 2015;33(10): 993-1011. doi: 10.1007/s40273-015-0312-9.
5. Acquadro C, Berzon R, Dubois D, et al. Incorporating the patient's perspective into drug development and communication: an ad hoc task force report of the Patient-Reported Outcomes (PRO) Harmonization Group meeting at the Food and Drug Administration, February 16, 2001.Value Health. 2003, 6(5):522-531. doi: 10.1046/j.1524-4733.2003.65309.x.
6. European Medicines Agency. Appendix 2 to the guideline on the evaluation of anticancer medicinal products in man -The use of patient-reported outcome (PRO) measures in oncology studies-. https://www.ema.europa.eu/en/appendix-2-guideline-evaluation-anticancer-medicinal-products-man-use-patient-reported-outcome-pro.
7. Williamson PR, Altman DG, Bagley H, et al. The COMET Handbook: version 1.0. Trials. 2017;18(Suppl 3):280. doi 10.1186/s13063-017-1978-4
8. Snyder CF, Aaronson NK, Choucair AK, et al. Implementing patient-reported outcomes assessment in clinical practice: a review of the options and considerations. Qual Life Res. 2012;21(8):1305-14. doi: 10.1007/s11136-011-0054-x.
9. Walton MK, Powers JH, Hobart J, et al. Outcome Assessments: Conceptual Foundation–Report of the ISPOR Clinical Outcomes Assessment – Emerging Good Practices for Outcomes Research Task Force. Value Health. 2015;18(6):741-52. doi: 10.1016/j.jval.2015.08.006.
10. US Food and Drug Administration. Drug Development Tool (DDT) Qualification Programs. https://www.fda.gov/drugs/development-approval-process-drugs/drug-development-tool-ddt-qualification-programs.
11. EUnetHTA 21. EUnetHTA 21 – Individual Practical Guideline Document, D4.4 – OUTCOMES (ENDPOINTS). https://www.eunethta.eu/wp-content/uploads/2023/01/EUnetHTA-21-D4.4-practical-guideline-on-Endpoints-v1.0.pdf.
12. US Food and Drug Administration. Qualified Clinical Outcome Assessments (COA).　https://www.fda.gov/drugs/clinical-outcome-assessment-coa-qualification-program/qualified-clinical-outcome-assessments-coa.
13. Core Outcome Measures in Effectiveness Trials. Core Outcome Measures in Effectiveness Trials(COMET).　https://www.comet-initiative.org/.
14. US Department of Health and Human Services. FDA Patient-Focused Drug Development Guidance Series for Enhancing the Incorporation of the Patient’s Voice in Medical Product Development and Regulatory Decision Making. https://www.fda.gov/drugs/development-approval-process-drugs/fda-patient-focused-drug-development-guidance-series-enhancing-incorporation-patients-voice-medical.
15. Mayo NE, Figueiredob S, Ahmed S, et al. Montreal Accord on Patient-Reported Outcomes (PROs) use series - Paper 2: terminology proposed to measure what matters in health. J Clin Epidemiol. 89,119-124. doi: 10.1016/j.jclinepi.2017.04.013.
